# Editorial: Microbiology and pathogenesis of Chlamydia, Coxiella, and Rickettsia

**DOI:** 10.3389/fcimb.2024.1445682

**Published:** 2024-06-27

**Authors:** Matteo Bonazzi

**Affiliations:** Institut de Recherche en Infectiologie de Montpellier (IRIM), Centre Nationale de la Recherche Sicentifique (CNRS), Université de Montpellier, Montpellier, France

**Keywords:** host/pathogen interactions, obligate intracellular pathogens, Chlamydia, Coxiella burneti, Rickettsia

This Research Topic sheds light on the complex interplay between obligate intracellular pathogens and their hosts, the innovative methods driving new discoveries, and the ongoing challenges in combatting these infections. If we consider that eukaryotic cells as we know them have been shaped by the co-evolution with symbiotic microbes, bacterial adaptation to an intracellular lifestyle is very ancient. Initially internalised as nutrients by grazing unicellular organisms, some microbes have been gradually selected for their capacity of surviving and replicating within phagocytic cells. Accessing an intracellular environment presents several advantages over the extracellular lifestyle. Inside eukaryotic cells, microbes can escape harsh conditions encountered in the extracellular environment including the humoral immune system. Furthermore, the cytoplasm and the lumen of intracellular vesicles are nutrient-rich environments that can be exploited by microbes. Finally, eukaryotic cells can transport microbes over long distances, thereby promoting their dissemination and granting access to organs that are distant from the initial point of access. Intracellular lifestyle turned out to become such an advantage for many microbes, that these evolved means to ease their internalisation, either by exposing proteins that mimic ligands for eukaryotic cell-surface receptors or by injecting effector proteins into the cytoplasm of host cells to trigger local plasma membrane remodelling mimicking phagocytosis and micropinocytosis in non-phagocytic cells.

Over the course of evolution, some intracellular microbes have adapted so well to the intracellular environment to gradually shed genes essential for an extracellular lifestyle, including DNA regions encoding entire metabolic pathways, becoming strictly dependent on their host to scavenge energy, nutrients and metabolites required for replication. Research into these processes not only enhances our understanding of bacterial survival strategies but also identifies potential targets for therapeutic intervention. Recent advances on this topic are reviewed here by Mandel et al. (Mandel et al.). Curiously, among genes shed during intracellular adaptation are also components of the divisome and elongasome, that regulate binary cell division. This suggests that plasticity in bacteria cell division processes is also part of intracellular adaptation (Harpring and Cox).

Despite the interest that infections by these pathogens have raised, an obligate intracellular lifestyle historically imposed important constraints in our capacity to identify, validate and investigate their virulence determinants and adaptation strategies. Advances in whole genome sequencing and comparative genomics has provided unprecedented insights into their virulence, evolution, and phylogeny of obligate intracellular pathogens, revealing the genetic basis for pathogenicity and host specificity. As illustrated by Luu et al., these approaches allowed the identification of conserved and unique genetic elements that could serve as targets for diagnostics and therapeutics (Luu et al.). In parallel, innovations in genetic systems have revolutionized the study of obligate intracellular human-pathogenic bacteria. Fisher and Beare present advances in the development of new tools for genetic manipulation, including CRISPR-Cas systems and transposon mutagenesis, which have facilitated functional genomics studies, enabling researchers to dissect the roles of specific genes in pathogenesis and survival (Fisher and Beare). These important breakthroughs fostered remarkable progress in the study of obligate intracellular bacteria.

Key to the virulence of obligate intracellular vacuolar pathogens, such as *Chlamydia* and *Coxiella*, is the establishment and maintenance of a niche within host cells. These compartments, that only exist in the context of infections, are composed of host and microbial proteins, opening fascinating research avenues in cell biology and host/pathogen interactions. The biogenesis of intracellular replicative niches perfectly represents the sophisticated interactions with host cell signalling pathways and membrane trafficking and largely rely on microbial effector proteins. These are translocated from the cytosol of bacteria to that of the host cell by specific nanomachines, the secretion systems. Understanding these mechanisms is crucial for developing targeted therapies. Here, Clemente et al., Jury et al. and Bui et al. illustrate the complexity of host/pathogen interactions functional for niche biogenesis and infection and shed light on new advances on the function of Type 1 Secretion Systems used by Rickettsiales, highlighting newly identified immunoreactive substrates (Bui et al.; Clemente et al.; Jury et al.).

A logical consequence of adapting an obligate intracellular lifestyle is the evolution of strategies to evade and/or dampen innate immune recognition by the host. These bacteria can avoid detection and destruction by modulating host cell processes, such as preventing the fusion of phagosomes with lysosomes, thereby avoiding degradation. They also interfere with host signalling pathways to dampen inflammatory responses, inhibit apoptosis, and subvert autophagy. Additionally, these pathogens can manipulate host immune responses by altering cytokine production and immune cell recruitment, ensuring a favourable intracellular environment for their proliferation while evading the host’s innate defences. Londoño et al. report intriguing examples of these strategies deployed by *Rickettsia* species and *Anaplasma phagocytophylum* (Londoño et al.). Regardless of their capacity of inhibiting the innate immune response and hijack membrane traffic to generate an intracellular niche compatible with replication, intravacuolar pathogens remain exposed to numerous host-specific stresses, including nutrient limitations. To overcome such challenges, bacteria evolved the capacity of forming persistent forms. Importantly, development of persistence is associated to antibiotic therapy failure and chronic infections. In the context of *Chlamydia* infections, understanding persistence mechanisms is critical for addressing chronic and recurrent infections (Riffaud et al.). Importantly, these type of infections in the female genital tract can lead to fibrosis of the fallopian tubes, consistently linked to scarring, causing ectopic pregnancy or infertility. Recent studies report that this could be directly linked to *C. trachomatis* induced growth factor signalling and pro-fibrotic remodelling of the extracellular matrix (Caven and Carabeo). Additionally, *C. trachomatis* infection can cause epithelial cells to undergo an epithelial-to-mesenchymal transition, transforming into a myofibroblast-like phenotype (Caven and Carabeo). In addition, a study by Ardizzone et al. highlights the complex interplay between *C. trachomatis* infection, the vaginal microbiota, and bacterial vaginosis (BV). It examines how the burden of *C. trachomatis* correlates with changes in the vaginal microbiota and the impact of metronidazole treatment on this relationship (Ardizzone et al.). These findings underscore the importance of considering the vaginal microbiota in managing *Chlamydia* infections and suggest potential avenues for improving treatment outcomes.

In conclusion, the study of obligate intracellular bacteria continues to reveal new dimensions of their complex biology and interactions with host organisms. However, significant challenges remain, particularly in the areas of persistence, immune evasion, and vaccine development. Advances in genetic tools, genomics, and an improved understanding of host-pathogen dynamics are paving the way for innovative treatments and vaccines, which remains a significant challenge. New tools and approaches are being employed to develop vaccines against these pathogens. Recent progress in this area reviewed by Van Schaik et al. offers hope for effective vaccines that could prevent infections and reduce the global burden of diseases caused by these elusive bacteria (Schaik et al.). Continued research and collaboration are essential to overcome these hurdles and combat the diseases caused by these formidable pathogens.

## Author contributions

MB: Writing – original draft, Writing – review & editing.

